# Correlation of urinary loss rate after catheter removal and long‐term urinary continence after robot‐assisted laparoscopic radical prostatectomy

**DOI:** 10.1111/iju.14488

**Published:** 2021-01-28

**Authors:** Tomoyuki Tatenuma, Kazuhide Makiyama, Yusuke Ito, Kentaro Muraoka, Hisashi Hasumi, Narihiko Hayashi, Keiichi Kondo, Noboru Nakaigawa, Masahiro Yao

**Affiliations:** ^1^ Department of Urology Yokohama City University Hospital Yokohama Kanagawa Japan

**Keywords:** catheter removal, continence, prostate cancer, robot‐assisted radical prostatectomy, urine loss rate

## Abstract

**Objectives:**

To assess the correlation of urine loss rate after catheter removal with long‐term continence after robot‐assisted radical prostatectomy.

**Methods:**

We enrolled 163 patients on whom robot‐assisted radical prostatectomy was carried out and whose urine loss rate we were able to evaluate after catheter removal. Urinary incontinence was evaluated from immediately after removal of the catheter to the date of discharge, and at 1, 3, 6 and 12 months after surgery. Urine loss rate was defined as the urine loss volume divided by the total urine volume.

**Results:**

The continence rates of patients with ≤1% urine loss rate on the day of catheter removal were 100% at 6 and 12 months after surgery. A multivariate analysis proved that ≤10% urine loss rate on the day of catheter removal was a significant predictor of continence at 3 months after surgery. Furthermore, the continence rate at 12 months of patients who did not achieve ≤10% urine loss rate on the day of catheter removal was 79.5%. Among them, the continence rate at 12 months of patients who achieved ≥15% urine loss rate improvement from the day of catheter removal to the next day was 95.2%; the factor differed significantly between the continence and incontinence groups at 12 months after surgery.

**Conclusions:**

The urine loss rate on the day of catheter removal is significantly related to the acquisition of urinary continence. Furthermore, our findings suggest that long‐term urinary continence can be expected, even in the event of poor urine loss rate on the day of catheter removal, if it improves on the next day.

**Abbreviations & Acronyms**
BMI: body mass index
LRP: laparoscopic radical prostatectomy
PSA: prostate‐specific antigen
RARP: robot‐assisted radical prostatectomy
RRP: radical retropubic prostatectomy
ULR: urine loss rate

## Introduction

RARP is more advantageous in urinary continence than LRP and RRP.^1^, ^2^ Urinary incontinence remains a significant complication that lowers the quality of life of patients who received radical prostatectomy. Various predictors of urinary continence have been reported; for example, age, obesity, length of the membranous urethra, anastomotic stricture, the experience of the surgeon, neurovascular bundle preservation, large prostate volume, obstructive urinary symptoms and preservation of the bladder neck.^3^ Among the predictors, it has been reported that the ULR immediately after catheter removal can predict long‐term urinary continence in RRP and LRP.^4^, ^5^ However, there are few reports of the correlation of ULR after catheter removal and urinary continence.

In the present study, we investigated whether ULR is associated with long‐term urinary continence in RARP.

## Methods

### Patients

We carried out 504 RARPs on patients with prostate cancer between October 2014 and October 2018 in Yokohama City University Hospital, Yokohama, Kanagawa, Japan. Almost all of the patients measured total urine volume and urine loss volume on the day of catheter removal. We confirmed the urination data of 163 of those patients, retrospectively. This study was approved by the ethics committee of our institution.

RARP was carried out by six surgeons. The surgical procedure was unified among the surgeons. We carried out bladder neck preservation if prostate cancer did not exist near the bladder neck. We performed anterior and posterior reconstruction. The choice of nerve‐sparing was decided by patients’ and surgeons’ preference.

### Urine loss rate

The urethral catheter was generally removed 5 or 6 days after surgery. After its removal, micturition volumes and urine loss volumes in the pads for each void were measured every day until discharge. The ULR was defined as the urine loss volume divided by the total urine volume. ULR was categorized as <1%, 1–10%, 10–50% and >50%.

### Continence definition

Urinary continence was evaluated at 1, 3, 6 and 12 months after surgery. Urinary continence was limited to one safety pad. Continence rates were summarized by ULR category.

### Significant factors for continence

We investigated significant factors for continence at 3 months after surgery, such as patient age, BMI, initial PSA, clinical T stage (cT1c *vs* cT2 or more), D’Amico classification risk (low *vs* intermediate *vs* high), console time, blood loss, pathological stage (pT2 *vs* pT3 or more), Gleason score (6 *vs* 7 *vs* ≥8), nerve‐sparing and ULR on the day of catheter removal. Furthermore, among patients with poor ULR on the day of catheter removal, we investigated significant factors for continence at 12 months after surgery, including similar factors and improvement in the ULR from the day of catheter removal to the next day.

### Statistical analysis

The data were analyzed using spss, version 20 (IBM Corporation, Armonk, NY, USA). For statistical analysis, univariate analysis was used to compare continence and incontinence groups. An unpaired *t*‐test was used for continuous variables, and a χ^2^‐test was used for categorical variables. Logistic regression analysis was used for multivariate analysis to identify the factors of continence. *P*‐values of <0.05 were considered to show statistical significance.

## Results

Continence rates at 1, 3, 6 and 12 months after surgery depend on the ULR immediately after catheter removal.

Table 1 shows that the continence rates at 1, 3, 6 and 12 months after surgery depend on the ULR on the day of catheter removal. Continence rates of all patients at 1, 3, 6 and 12 months after surgery were 20.4%, 54.8%, 75.9% and 85.7%, respectively. A patient with a ULR of ≤1% on the day of catheter removal could achieve 100% continence at 6 months after surgery. A patient with a ULR of 1–10% on the day of catheter removal could achieve >80% continence after 6 months.

**Table 1:** Relationship of urine loss rate after catheter removal and continence rate at 1, 3, 6 and 12 months after RARP

	ULR ≤1%	ULR 1–10%	ULR 10–50%	ULR >50%	All patients
1 month	69.6% (16/23)	28.2% (11/39)	7.3% (4/55)	4.5% (2/45)	20.4% (33/162)
3 months	82.6% (19/23)	70.3% (26/37)	48.1% (25/52)	35.7% (15/43)	54.8% (85/155)
6 months	100% (21/21)	80.6% (25/31)	77.1% (37/48)	60% (24/41)	75.9% (107/141)
12 months	100% (20/20)	92.3% (24/26)	82.5% (33/40)	78.1% (25/33)	85.7% (102/119)

### Significant factors for continence at 3 months after surgery

Table 2 compares the continence and incontinence groups at 3 months after surgery. Console time and ULR differ significantly between the two groups. A multivariate analysis was carried out, including these factors (Table 3). A ULR of ≤10% on the day of catheter removal was an independent significant factor for continence at 3 months after surgery.

**Table 2:** Comparison of clinical and pathological characteristics between continence and incontinence patients at 3 months after RARP

	Continence at 3 months (*n* = 85)	Incontinence at 3 months (*n* = 70)	*P*‐value
Age, years (mean ± SD)	67.5 ± 6.1	68.0 ± 5.4	0.57
BMI (mean ± SD)	23.6 ± 2.8	23.9 ± 3.0	0.53
IPSS before surgery			
Symptoms (mean ± SD)	8.6 ± 6.3	9.03 ± 6.3	0.68
Quality of life (mean ± SD)	3.4 ± 1.6	3.3 ± 1.4	0.64
OABSS before surgery (mean ± SD)	3.7 ± 2.6	3.6 ± 2.5	0.74
Initial PSA ng/mL (mean ± SD)	10.6 ± 7.5	10.0 ± 7.0	0.63
Clinical stage			0.60
T1	23.5% (20)	20% (14)	
T2 or more	76.5% (65)	80% (56)	
D’Amico			0.95
Low	12.9% (11)	12.9% (9)	
Intermediate	44.7% (38)	44.3% (31)	
High	42.4% (36)	42.9% (30)	
Console time, min (mean ± SD)	175 ± 45	194 ± 43	0.01
Blood loss, mL (mean ± SD)	155 ± 426	171 ± 226	0.78
Prostate volume, g (mean ± SD)	48.3 ± 16.5	50.7 ± 16.6	0.37
Pathological stage			0.30
T2	68.2% (58)	75.7% (53)	
T3 or more	31.8% (27)	24.3% (17)	
Gleason score			0.24
6	5.1% (4/79)	2.9% (2/70)	
7	64.6% (51/79)	78.6% (55/70)	
8–10	30.4% (24/79)	18.6% (13/70)	
Nerve‐sparing (+)	9.4% (8)	17.1% (12)	0.15
Unilateral	6	12	
Bilateral	2	0	
Nerve‐sparing (−)	90.6% (77)	82.9% (58)	
ULR ≤10% at day 1	52.9% (45)	21.4% (15)	<0.001

**Table 3:** Multivariate analysis of urinary continence at 3 months after RARP

	Odds ratio	95% CI	*P*‐value
Age	0.960	0.885–1.04	0.31
BMI	0.901	0.780–1.04	0.15
D’Amico	1.010	0.523–1.96	0.96
Console time	0.996	0.986–1.00	0.35
Nerve‐sparing	0.930	0.189–4.59	0.92
ULR ≤10% at day 1	2.790	1.010–7.67	0.04

### Significant factors for continence at 12 months after surgery in cases of poor ULR on the day of catheter removal

For patients with ULRs of 10–50% and >50% on the day of catheter removal, continence rates at 12 months after surgery were 82.5% and 78.1% (Table 1). These continence rates are relatively poor. Among patients with a ULR of >10%, the continence rate at 12 months of the patients who showed a ≥15% ULR improvement from the day of catheter removal to the next day was 95.2% (Table 4). Console time and improvement in ULR of >15% from the day of catheter removal to the next day differed significantly between the continence and incontinence groups at 12 months after surgery (Table 5). Multivariate analysis showed that console time was an independent significant factor for continence at 12 months after surgery (Table 6). The *P*‐value of improvement in ULR of >15% from the day of catheter removal to the next day was 0.053.

**Table 4:** Relationship of ULR improvement from the day of catheter removal to the next day and continence rate at 3, 6 and 12 months after RARP among patients with a ULR of >10% after catheter removal

	<15%	≥15%
Continence at 3 months	40.9% (18/44)	35.8% (8/23)
Continence at 6 months	63.6% (28/44)	78.3% (18/23)
Continence at 12 months	71.8% (28/39)	95.2% (20/21)

**Table 5:** Comparison of clinical and pathological characteristics between continence and incontinence patients at 12 months after RARP in cases of poor ULR on the day of catheter removal

	Continence at 12 months (*n* = 58)	Incontinence at 12 months (*n* = 15)	*P*‐value
Age, years (mean ± SD)	68.3 ± 5.5	70.0 ± 4.1	0.26
BMI (mean ± SD)	23.5 ± 2.9	23.9 ± 2.2	0.53
IPSS before surgery			
Symptoms	8.5 ± 6.1	9.8 ± 6.7	0.50
Quality of life	3.5 ± 1.7	3.2 ± 1.1	0.93
OABSS before surgery (mean ± SD)	3.8 ± 2.7	3.2 ± 2.8	0.44
Initial PSA ng/mL (mean ± SD)	10.2 ± 6.1	12.3 ± 8.5	0.28
Clinical stage			0.21
T1	20.7% (12)	6.7% (1)	
T2 or more	79.3% (46)	93.3% (14)	
D’Amico			0.30
Low	8.6% (5)	12.9% (9)	
Intermediate	41.4% (24)	44.3% (31)	
High	50% (29)	42.9% (30)	
Console time, min (mean ± SD)	189 ± 46	225 ± 48	0.01
Blood loss, mL (mean ± SD)	182 ± 517	160 ± 176	0.87
Prostate volume, g (mean ± SD)	48.3 ± 14.0	49.1 ± 17.7	0.64
Pathological stage			0.51
T2	69.0% (40)	60% (9)	
T3 or more	31.0% (18)	40% (6)	
Gleason score			0.91
6	5.1% (3/56)	0% (0/15)	
7	64.6% (36/56)	73.3% (11/15)	
8–10	30.4% (24/79)	26.7% (4/15)	
Nerve‐sparing (+)	10.3% (6)	0% (0)	0.53
Unilateral	6	0	
Bilateral	0	0	
Nerve‐sparing (−)	89.7% (52)	100% (15)	
(ULR at day 1 – ULR at day 2) ≥15%	41.6% (20/48)	8.3% (1/12)	0.03

**Table 6:** Multivariate analysis of urinary continence at 12 months after RARP in cases of poor ULR on the day of catheter removal

	Odds ratio	95% CI	*P*‐value
Age	0.869	0.716–1.06	0.15
BMI	0.923	0.686–1.24	0.59
D’Amico	0.752	0.206–2.74	0.66
Console time	0.976	0.957–0.996	0.01
Nerve‐sparing	–	0.000–Inf	0.99
(ULR at day 1 – ULR at day 2) ≥15%	10.100	0.970–105	0.053

## Discussion

A systematic review showed that RARP improved the functional outcomes of urinary continence, compared with RRP or LRP. Reported incontinence rates at 12 months ranged from 8% to 11%, with a mean value of 9%.^1^ Despite improved continence rates, urinary incontinence remains an important concern for patients. To predict urinary continence, a comprehensive prediction model was reported. This report showed some risk factors, such as age‐adjusted Charlson Comorbidity Index, International Index of Erectile Function‐5, prostate volume, nerve‐sparing status and 24‐h urine loss at 1 month after RARP.^6^ Post‐surgery, patients can be relieved or can start medication early if their urinary continence can be accurately predicted early. Therefore, a prediction on the day of catheter removal is useful.

It was reported that urine loss volume on the day of catheter removal after RRP was significantly related to urinary incontinence.^7^, ^8^ Sato *et al*. reported that all patients achieved urinary continence by 12 months after RRP if the ULR was ≤1% within 7 days of catheter removal, or ≤5% within 3 days of catheter removal.^5^ Ates *et al*. reported that ULR on the day of catheter removal after LRP was related to the period required to achieve urinary continence.^4^ In the present study, a ULR of ≤10% on the day of catheter removal was an independent significant factor for continence at 3 months after RARP. This suggests that ULR on the day of catheter removal is also a useful predictor of urinary continence after RARP. Furthermore, ULR can predict urinary continence at just 3 months. For example, patients who will achieve urinary continence early can plan to return to work early.

Conversely, the continence rates of patients who did not have ULRs of <10% were relatively poor. Among them, patients with a >15% ULR improvement from the day of catheter removal to the next day tended to achieve urinary continence at 12 months after surgery. It was suggested that long‐term urinary incontinence could be expected if the ULR was poor on the day of catheter removal, even if it improved the next day. Ates *et al*. reported that ULR on the last day of hospitalization was more significantly correlated with time to continence than first‐day ULR.^4^ On the day of catheter removal, patients might have an overactive bladder and sphincter weakness. Therefore, a poor ULR on the day of catheter removal might not show the true continence potential.

The present study showed console time was important factor for urinary continence. In particular, console time was an independent significant factor for continence at 12 months after surgery in cases of poor ULR on the day of catheter removal. Console time might depend on the skill of the surgeons. The surgical technique of urinary continence is not different among the surgeons because of the standardization of surgical techniques. Nevertheless, experienced surgeons and experts are likely to carry out the surgical technique of urinary continence more accurately and quickly. It was reported that increased experience with RARP resulted in improvements in urinary continence.^9^ RARP was carried out by six surgeons in the present study. Among them, two urologists were experienced surgeons who had operated on >150 cases. In contrast, three of the surgeons had operated on <30 cases. We excluded the factor of surgeons, because there was a large difference in the number of cases experienced.

There were some limitations to the present study. First, the number of patients was relatively small. We carried out >500 RARPs in this period. However, some patients could not keep urination diaries accurately or a leak was reported in collecting records. As a result, we were able to enroll just one‐third of those RARP patients. In addition, many patients were discharged the day after catheter removal. Therefore, the number of patients who could keep a urination diary on that day was even smaller. Second, nerve‐sparing was carried out on just 13% of patients. This procedure is reported to be useful for urinary continence.^10^, ^11^ However, the present study did not show nerve‐sparing to be associated with urinary continence, probably due to the small number of nerve‐sparing cases. We might have to extend adaptation widely for better urinary continence.

Despite such limitations, the present study showed that ULR on the day of catheter removal is significantly related to the acquisition of urinary continence. Furthermore, it suggested that long‐term urinary continence can be expected, even in the event of poor ULR on the day of catheter removal, if it improves on the next day.

## Conflict of interest

None declared.
